# High plant protein intake decreases all-cause mortality in the “Seguimiento Universidad de Navarra” (SUN) cohort European journal of nutrition

**DOI:** 10.1007/s00394-026-04037-0

**Published:** 2026-08-01

**Authors:** Ainara Martínez-Tabar, Miguel Ruiz-Canela, Vanessa Bullón-Vela, Carmen de la Fuente-Arrillaga, Carmen Sayón-Orea, Miguel Ángel Martínez-González, Maira Bes-Rastrollo

**Affiliations:** 1 Department of Preventive Medicine and Public Health, University of Navarra, Institute for Nutrition and Health (INS), Pamplona, Spain; 2 IdiSNA (Navarra Medical Research Institute), Pamplona, Spain; 3 CIBER Physiopathology of Obesity and Nutrition (CIBEROBN), Madrid, Spain

**Keywords:** Mortality, Plant protein, Animal protein, Prospective, Cohort

## Abstract

**Purpose:**

Prospective cohort studies analyzing the effect of plant versus animal protein intake on mortality in relatively young individuals from Mediterranean populations are limited. Moreover, a limited number of studies have performed repeated measurements of protein intake. We evaluated the relationship between plant versus animal protein intake (at baseline and at the ten-year follow-up) and mortality in the “Seguimiento Universidad de Navarra” (SUN) project.

**Methods:**

The SUN project is a prospective, multi-purpose, dynamic cohort study of Spanish university graduates. Plant and animal protein intake were assessed using a semi-quantitative food frequency questionnaire (FFQ) previously validated in Spain. Participants were divided into quartiles based on their intake of protein. Cox regression models were used, with the first quartile serving as the reference category.

**Results:**

A total of 17,989 subjects (10,961 women and 7028 men) were included in the analysis. During 251.363 person-years of follow-up (median follow-up time: 12 years), 460 deaths were identified. The mean age at baseline was 38 years with a standard deviation of 12 years. Participants in the highest quartile of plant protein intake had a 35% lower risk of all-cause mortality compared to those in the lowest quartile after adjusting for potential confounders [hazard ratio (HR): 0.65 (95% CI: 0.45–0.93), *p* for trend = 0.009]. No significant association was found between animal protein intake and mortality [HR: 0.94 (95% CI: 0.70–1.26); *p* for trend = 0.555] nor between plant protein intake and cardiovascular or cancer mortality.

**Conclusion:**

Plant protein intake was inversely associated with all-cause mortality in a Mediterranean population. Animal protein intake was not associated with total mortality.

**Supplementary Information:**

The online version contains supplementary material available at 10.1007/s00394-026-04037-0.

## Introduction

In recent years, plant-based diets have been gaining popularity [1]. These diets are associated with a reduced risk of cardiovascular disease [2, 3] and better cardiometabolic profile [4, 5]. Additionally, plant-based diets have been linked to lower mortality rates from all causes, including cardiovascular disease [6]. In a plant-based diet, cereals, legumes, and nuts are the main plant protein rich foods, whereas in an omnivorous diet animal protein sources are, meat, fish, dairy, and eggs. Plant and animal proteins have different combinations of amino acids, resulting in varied effects on health [7]. Plant proteins typically contain lower levels of essential amino acids such as methionine and lysine, but higher levels of non-essential amino acids like arginine and glycine. Some studies suggested that arginine and glycine intake could be associated with a reduced risk of cardiovascular disease and mortality [8, 9].

Previous studies that evaluated the association between animal versus plant protein intakes and risk of all-cause, cardiovascular disease and cancer mortality described inconsistent findings. In the Nurses´ Health and Health Professionals Follow-up Study, Song et al. found that higher plant protein intake was associated with a lower risk of all-cause and CVD mortality [10]. Similarly, Kelemen et al. [11] and Budhathoki et al. [12] showed that plant protein intake was associated with lower mortality. In contrast, no association was found in the PREvention with MEditerranean DIet (PREDIMED) study [13] and the Rotterdam prospective cohort study [14].

The role of the animal protein intake on mortality was evaluated, in the Nurses´ Health study and Health Professionals Follow-up Study [10] and PREDIMED study [13]. These studies showed a positive association between animal protein intake and mortality. However, in the studies conducted by Budhathoki et al. in 2019 [12], Papanikolaou et al. [15] in 2019 and Huang et al. in 2020 [16] no association between animal protein intake and mortality was described.

The evidence regarding the effects of plant protein intake and animal protein intake on long-term health remains controversial. Furthermore, to our knowledge, no large prospective cohort study has been conducted in relatively young individuals from Mediterranean populations. Moreover, as dietary habits change over time, it is crucial to assess how these changes influence mortality. However, most available studies have only evaluated protein intake at baseline. Therefore, the aim of this study was to evaluate the association between plant versus animal protein intake (at baseline and at the ten-year follow-up) and mortality in a Mediterranean population of relatively young adults, using data from the “Seguimiento Universidad de Navarra” (SUN) cohort.

## Materials and methods

### Study design and population

The SUN Project is a prospective, multipurpose, and dynamic Spanish cohort formed of university graduates. It was designed to evaluate the effect of diet and lifestyle on the prevention of several non-communicable diseases and mortality. The eligibility criteria were being a university graduate and having Spanish nationality. Details about the design and methodology of the study have been previously reported [17]. Briefly, the recruitment of participants started in December 1999 and is permanently ongoing. Information is gathered every two years through mailed or web-based questionnaires. Participants provide information on diet, lifestyle, risk factors and report diagnosis of new diseases. Volunteers indicated their implied consent to participate in the study by filling out and submitting the first self-administered questionnaire. All prospective participants were informed of their right to refuse participation or withdraw their consent at any time without facing any repercussions. To safeguard the confidentiality and privacy of participants, the data were pseudonymized, assigning each participant a unique study code number. This study adhered to the principles outlined in the Declaration of Helsinki. The voluntary completion of the baseline questionnaire was interpreted as an expression of informed consent. The Research Ethics Committee of the University of Navarra approved this method for obtaining participants’ informed consent, with approval granted by the Human Research Ethical Committee at the University of Navarra (2001/30).

### Dietary assessment

Protein intake was assessed at baseline and after 10 years of follow-up with a semi-quantitative 136-item food-frequency questionnaire (FFQ) validated and repeatedly re-evaluated in Spain [18, 19]. The validated FFQ gathers the frequencies of food consumption in nine categories ranging from “never or almost never” to “≥6 times a day”. To estimate protein intake, we multiplicated frequency of consumption by the protein content of a standard portion size specified for each food. We used the available data from the food composition tables for Spain [20, 21].

Plant protein intake was calculated including protein from all plant-based foods (fruits and vegetables, legumes, cereals, nuts, dried fruits, coffee, alcoholic beverages and jams). We calculated the intake of animal protein including protein from all animal foods (dairy, red meat, white meat, processed meat, fish and seafood, egg and other animal foods such as mayonnaise). In the case of foods with different protein sources (pizza, croquettes, pies, pastries, bakery and cakes) they were divided according to their ingredients using Spanish recipes [22]. We assigned the amount of protein from each ingredient to the suitable protein group.

### Outcome assessment

Study outcome was all-cause mortality. Continuous contact with participants was maintained through postal address, email and telephone. More than 85% of deaths were identified by reports from next of kin, work colleagues, and the postal system. To confirm the rest of the deaths, the Spanish National Death Index was checked at least once a year to update vital status and identify causes of death, classified according to the 10th Revision of the International Classification of Diseases (ICD-10).

### Assessment of other variables

From the baseline questionnaire, we also gathered information about sociodemographic and lifestyle variables including age, sex, educational level, marital status, smoking status, alcohol intake, total energy intake and physical activity [23]. Body mass index (BMI) for each participant was calculated using self-reported height and weight values at baseline, which were previously validated in one subsample of the SUN cohort [24]. Mediterranean diet adherence was assessed using the score proposed by Trichopoulou et al. [25]. Total energy and nutrient intake were estimated from the FFQ administered at baseline and after 10 years of follow-up using Spanish food composition Tables [20, 21]. Finally, we also collected medically diagnosed conditions, such as cancer, cardiovascular disease, diabetes and hypertension.

### Statistical analysis

Participants were divided into quartiles according to their plant and animal protein intake. We described baseline characteristics of the participants across the four categories of plant and animal protein intake. We used percentages for categorical variables and mean and standard deviations for quantitative variables.

Plant and animal protein intake was adjusted for total energy intake using the residuals method separately for men and women proposed by Willett et al. [26].

We ran Cox regression models to assess the association between energy adjusted quartiles of plant and animal protein intake at baseline and all-cause mortality and cause specific mortality.

For each participant, we calculated person-years of follow-up from the date of fulfillment of the baseline questionnaire to the date of death or date when the last follow-up questionnaire was completed, whichever came first. Hazard ratios (HR) and their 95% confidence interval (CI) were calculated considering the lowest quartile of protein intake as the reference category and with age as underlying time-variable (birth date as origin).

An age-and sex-adjusted model and two multivariable Cox regression models were used to assess the association between protein intake and all-cause mortality and cause specific mortality. Model 1 was additionally adjusted for BMI (kg/m^2^, linear and quadratic terms, continuous), educational level (graduate, postgraduate and doctorate), marital status (married, yes/no), smoking status (never, current, former smoker), total cumulative exposure to cigarette smoking (pack-years, four categories), physical activity (METs-h/week, continuous), total energy intake (five categories), following a special diet (yes/no), snacking between meals (yes/no), Trichoupolou MeDiet score (three categories), alcohol intake (g/d, continuous), prevalent hypertension (yes/no) and prevalent hypercholesterolemia (yes/no). Model 2 was additionally adjusted for animal protein intake (g/d, continuous) when the assessed exposure was plant protein and for plant protein intake (g/d, continuous) when the assessed exposure was animal protein, monounsaturated fatty acids (g/d, continuous), polyunsaturated fatty acids (g/d, continuous), saturated fatty acids (g/d, continuous) and trans fatty acids (g/d, continuous). Analyses were stratified by recruitment period and by age group (10-year periods). Tests of linear trend across quartiles of plant and animal protein intake were conducted assigning the median value to each quartile of protein intake and introducing it in the model as a continuous variable. Multivariate Cox regression models were conducted using the cumulative means of plant and animal protein intake from baseline and 10-year follow-up information.

We selected potential confounders based on the published scientific literature (findings from other cohort studies that have analysed the association between protein intake and all-cause mortality) rather than statistical associations detected in the sample [27] .

Although we adjusted for a significant number of potential confounders, we cannot rule out the possibility of residual confounding. Protein intake may be related to other aspects of diet or lifestyle. To assess this, we estimated the E value proposed by VanderWeele [28]. The E value represents the minimum strength of association on the risk ratio scale that an unmeasured confounder would need to have with both protein intake and mortality to fully explain away a specific exposure-outcome association, conditional on the measured covariates.

To assess the contribution of the protein of each food group to the total intake of plant and animal protein, we calculated the ratio between the protein of each food group divided by the total g/d of plant protein or animal protein multiplied by 100. We also conducted analyses to identify the sources of heterogeneity within food groups, specifically focusing on plant and animal proteins. Cumulative R-squared values were calculated from the nested regression analysis following stepwise selection [26].

Deaths occurring over time were described using Nelson-Aalen cumulative hazard curves. We conducted inverse probability weighting to adjust the Nelson-Aalen curves for potential confounders. The predictors to calculate the stabilized weights were sex, age, BMI, educational level, marital status, smoking status, total cumulative exposure to cigarette smoking, physical activity, total energy intake, following a special diet, snacking, Trichoupolou MeDiet score, alcohol intake, prevalent hypertension, prevalent hypercholesterolemia, animal protein intake, monounsaturated fatty acids, polyunsaturated fatty acids, saturated fatty acids and trans fatty acids.

Substitution analyses were conducted using multivariable Cox regression models to estimate the risk of total mortality when replacing 5% of energy from plant protein with an equivalent energy contribution from animal protein, total carbohydrates, or fats. Plant protein and its corresponding substituted macronutrient were included as continuous variables in the multivariate model. To determine the HR and 95% CI for isocaloric substitution, we used the difference in regression coefficients.

Multiplicative interactions between quartiles of protein intake and different sociodemographic and lifestyle-related variables were tested with a likelihood ratio test comparing the models with and without the interaction product term. Possible effect modification of protein intake by sex, age (< 55/ ≥55 years), BMI (< 25/ ≥ 25 kg/m^2^), smoking (never/former or never smoker), physical activity (above median/below median (18.5 METs-h/wk)), baseline adherence to Mediterranean Diet (0–4/5–9 points), animal protein intake (below/above the median (73 g/d)) if the exposure variable was plant protein, and plant protein intake (below/above the median (31 g/d)) if the exposure variable was animal protein were assessed.

Sensitivity analyses were also performed to assess the robustness of our findings under different scenarios: using the 5th and 95th percentiles as limits for total energy intake; excluding subjects following special diets at baseline; omitting deaths in the first 2 years of follow-up; and excluding deaths from cancer, and cardiovascular diseases once at a time. We additionally adjusted for carbohydrates intake, glycemic load, fiber intake, cereals, vegetables and legumes consumption in the case of plant protein intake and for animal protein intake was additionally adjusted for iron, processed and unprocessed red meat. In sensitivity analyses, we also included carbohydrate intake as an adjustment variable instead of fatty acid intake. Moreover, we calculated plant and animal protein intake as the percentage of energy from plant or animal protein in relation to total energy intake and use it as the exposure.

All *p* values presented are two-tailed and *p* values of less than 0.05 were considered statistically significant. Analyses were carried out using STATA/SE version 15.0 (StataCorp, College Station, TX, USA).

## Results

Up to May 2022, 23,133 participants had completed the baseline questionnaire of the SUN Project. For the present analysis, we excluded 234 participants with insufficient follow-up time to complete at least one follow-up questionnaire, 2123 subjects who were outside of predefined limits for baseline total energy intake (< 500 or > 3500 kcal/d for women, and < 800 or > 4000 kcal/d for men) [26] and 1188 subjects reporting a baseline medical diagnosis of cardiovascular disease (CVD), cancer or Type 2 Diabetes Mellitus (T2DM). In addition, 220 participants with no answer in 70 or more items of the Food Frequency Questionnaire (FFQ) and 1379 participants who were lost to follow-up were excluded (retention rate: 93%). Finally, 17,989 participants (10,961 women and 7028 men) were included in the analyses (Supplemental Fig. 1).

During 251.363 person-years of follow-up (median follow-up time: 12 years), 460 deaths were identified (227 cancer deaths and 86 cardiovascular disease deaths). Participants´ mean age (standard deviation) at baseline was 38 (12) years.

**Table 1:** Baseline characteristics [mean and standard deviation (SD), or %] of participants according to quartiles (Q) of animal and plant protein intake in the SUN Project

Characteristics	Quartiles of energy-adjusted plant protein intake				Quartiles of energy-adjusted animal protein intake			
	Q1	Q2	Q3	Q4	Q1	Q2	Q3	Q4
*n*	4498	4497	4497	4497	4498	4497	4497	4497
Total protein intake (g/d) ^**a**^	107.0	104.9	104.8	105.9	87.4	100.1	109.2	125.9
	(19.7)	(15.4)	(15.2)	(18.0)	(10.5)	(6.7)	(7.0)	(13.8)
Plant protein intake (g/d) ^**a**^	23.4	29.1	33.1	41.0	35.4	32.2	30.7	28.3
	(3.4)	(1.1)	(1.3)	(5.8)	(8.1)	(6.1)	(6.3)	(6.7)
Animal protein intake (g/d) ^**a**^	83.8	76.1	72.0	65.1	52.3	68.2	78.8	97.8
	(20.4)	(15.4)	(15.2)	(18.3)	(9.7)	(3.1)	(3.3)	(13.9)
Age (years)	34.8	36.5	38.1	40.3	38.8	37.2	37.0	36.8
	(10.7)	(11.1)	(11.7)	(12.7)	(12.0)	(11.5)	(11.6)	(11.7)
Female (%)	60.9	60.9	60.9	60.9	60.9	60.9	60.9	60.9
Married (%)	44.9	49.7	51.0	51.7	49.9	48.6	49.9	48.9
Years of university education	5.0	5.0	5.1	5.1	5.1	5.1	5.0	5.0
	(1.4)	(1.5)	(1.5)	(1.6)	(1.5)	(1.5)	(1.5)	(1.5)
**Smoking (%)**								
Never smoker	48.7	49.1	49.8	50.4	48.5	49.7	50.2	49.6
Current smoker	26.6	22.7	20.9	18.5	21.5	22.6	22.0	22.5
Former smoker	24.7	28.2	29.3	31.1	29.9	27.7	27.8	27.8
**Prevalent diseases at baseline (%)**								
Hypertension	8.2	8.5	9.8	11.7	9.8	8.6	9.8	9.7
Hypercholesterolemia	13.3	15.3	17.1	19.7	17.3	16.1	16.1	15.7
BMI (kg/m^2^)	23.4	23.5	23.5	23.5	23.1	23.3	23.5	23.9
	(3.5)	(3.4)	(3.4)	(3.5)	(3.5)	(3.4)	(3.4)	(3.6)
Physical activity (METs-h/week)	20.2	21.3	21.5	24.3	21.7	20.9	21.4	23.3
	(22.4)	(22.2)	(21.5)	(25.6)	(22.8)	(22.1)	(21.5)	(25.4)
Television viewing (h/days)	1.7	1.6	1.6	1.5	1.6	1.6	1.6	1.6
	(1.2)	(1.1)	(1.1)	(1.1)	(1.1)	(1.2)	(1.2)	(1.2)
Snacking between meals (%)	40.6	34.0	31.3	28.0	33.0	32.8	32.6	35.5
Follow a special diet (%)	5.6	6.8	7.7	10.3	7.0	6.1	7.1	10.3
Total energy intake (kcal/days)	2470	2257	2252	2426	2437	2276	2273	2421
	(595)	(586)	(607)	(612)	(644)	(600)	(582)	(584)
**Macronutrients**								
Carbohydrate intake (%E)	38.7	42.2	44.7	48.4	48.7	44.6	42.0	38.7
	(6.7)	(5.9)	(6.1)	(6.9)	(7.1)	(6.0)	(5.9)	(6.4)
Protein intake (%E)	18.2	18.3	18.3	18.1	15.0	17.4	19.1	21.6
	(3.4)	(3.0)	(3.1)	(3.2)	(1.9)	(1.6)	(1.9)	(2.7)
Fat intake (%E)	40.8	37.4	35.1	31.8	34.2	35.9	36.9	38.0
	(6.0)	(5.1)	(5.2)	(5.9)	(7.1)	(6.1)	(6.0)	(6.1)
SFA (%E)	14.9	13.1	11.9	10.1	10.9	12.2	13.0	13.9
	(3.0)	(2.5)	(2.4)	(2.5)	(2.9)	(2.7)	(2.8)	(3.3)
MUFA (%E)	17.7	16.2	15.3	13.9	15.2	15.7	16.0	16.2
	(3.8)	(3.2)	(3.2)	(3.3)	(4.2)	(3.5)	(3.4)	(3.3)
PUFA (%E)	5.6	5.3	5.1	4.9	5.4	5.2	5.2	5.0
	(1.7)	(1.5)	(1.4)	(1.5)	(1.9)	(1.5)	(1.4)	(1.3)
Total dietary fiber (g/d)	17.1	19.4	22.6	31.1	26.2	22.0	21.0	21.1
	(6.5)	(6.8)	(7.5)	(11.1)	(11.9)	(8.9)	(8.5)	(8.3)
Alcohol intake (g/d)	7.9	6.6	6.0	5.7	7.5	6.8	6.2	5.8
	(12.2)	(9.8)	(8.3)	(8.4)	(11.8)	(10.0)	(8.8)	(8.3)
**Mediterranean diet score**,** Trichopoulou (%)**								
Low (0–3)	64.5	45.6	26.9	10.3	25.7	37.3	41.8	42.6
Median (4–5)	30.2	41.1	46.4	37.9	40.4	38.1	37.1	40.0
High (6–9)	5.4	13.3	26.7	51.8	33.9	24.7	21.1	17.5

We used percentages for categorical variables and mean and standard deviation for quantitative variables

MET: metabolic equivalent of task, E: Energy, SFA: Saturated Fatty Acids, MUFA: Monounsaturated Fatty acids, PUFA: Polyunsaturated Fatty Acids, % E: percentage of energy

^**a**^ Adjusted for energy intake using the residual method

The baseline characteristics of participants according to quartiles of plant protein intake and animal protein intake are described in Table 1. Participants in the higher quartile of plant protein intake (median: 39.3 g/d) compared with those in the lower quartile (median: 24.3 g/d) were more likely to be older and had a higher prevalence of hypertension and hypercholesterolemia. In addition, they were less likely to be current smokers and snack between meals but were more likely to have greater adherence to the Mediterranean diet. As expected, participants with higher plant protein intake (Q4) reported higher carbohydrate and fiber intake and lower fat and saturated fatty acid intake. Participants with a higher intake of animal protein (median: 93.8 g/d), compared with those with a lower intake (median: 55.1 g/d), were younger, more likely to snack and had lower adherence to the Mediterranean Diet. They also had lower carbohydrate and fiber intake but higher total fat and saturated fatty acid intake.

**Table 2:** Percentage of each protein source to the total amount of animal and plant protein and sources of variability (cumulative R^2^) in the SUN (“Seguimiento Universidad de Navarra”) cohort

Animal protein sources	% of total protein source	Cumulative *R*^2^
Dairy products	27.0	0.33
Unprocessed red meat	19.1	0.55
White meat	12.9	0.75
Fish and seafood	18.5	0.87
Processed red meat	13.9	0.98
Eggs	5.0	0.99
Other animal-based foods	3.5	1.00

Plant protein sources	% of total protein source	Cumulative R^2^
Cereals	33.4	0.38
Vegetables	32.2	0.65
Legumes	15.3	0.87
Nuts	3.9	0.96
Fruits	7.9	0.99
Other plant-based foods	8.2	1.00

18% [18.3% (SD = 3.3%)] of total energy came from protein intake, of which 5.4% (SD = 1.3) came from plant protein and 12.9% (SD = 3.3) from animal protein. Table 2 shows the percentage of each protein source to the total amount of animal and plant protein and sources of variability (cumulative R^2^). The main animal protein sources were dairy products (27.0%), unprocessed red meat (19.1%), fish and seafoods (18.5%), processed red meat (13.9%) and white meat (12.9%). These five animal protein sources explained 98% of the between person-variability in animal protein intake. The major contributors to plant protein intake were cereals (33.4%), vegetables (32.2%), legumes (15.3%) and other plant-based foods (8.2%). Cereals, vegetables, legumes and nuts explained 96% of the between person-variability in plant protein intake.

**Table 3:** Cox proportional HRs and 95% CIs for all-cause mortality and cause specific mortality according to animal and plant protein intake

Quartiles of energy-adjusted plant protein intake					
	Q1	Q2	Q3	Q4	
Median (g/d)	**24.3**	**29.1**	**33.0**	**39.3**	
**All-cause mortality**					
N	4498	4497	4497	4497	***p*** ** for trend**
Deaths/person-years	110/60,088	120/59,408	110/58,209	120/57,161	
Age and sex adjusted	1.00	0.93	0.71	0.55	< 0.001
	(ref.)	(0.72–1.20)	(0.54–0.93)	(0.42–0.72)	
Model 1	1.00	0.98	0.80	0.63	0.002
	(ref.)	(0.75–1.27)	(0.60–1.08)	(0.46–0.86)	
Model 2	1.00	0.99	0.81	0.65	0.009
	(ref.)	(0.75–1.31)	(0.60–1.11)	(0.45–0.93)	
Repeated measurements of diet	1.00	0.98	0.81	0.67	0.012
	(ref.)	(0.72–1.31)	(0.59–1.11)	(0.47–0.96)	
**Cancer deaths**					
N	4441	4443	4442	4430	***p*** ** for trend**
Deaths/person-years	53/59,472	66/58,746	55/57,576	53/56,322	
Age and sex adjusted	1.00	1.02	0.71	0.52	< 0.001
	(ref.)	(0.71–1.48)	(0.49–1.05)	(0.35–0.77)	
Model 1	1.00	1.10	0.80	0.61	0.013
	(ref.)	(0.75–1.62)	(0.52–1.22)	(0.38–0.97)	
Model 2	1.00	1.16	0.85	0.67	0.039
	(ref.)	(0.78–1.71)	(0.54–1.32)	(0.40–1.13)	
Repeated measurements of diet	1.00	1.06	0.75	0.60	0.014
	(ref.)	(0.70–1.61)	(0.47–1.19)	(0.37–1.00)	
**Cardiovascular death**					
N	4405	4403	4406	4401	***p*** ** for trend**
Deaths/person-years	17/59,043	26/58,286	19/57,230	24/56,025	
Age and sex adjusted	1.00	1.29	0.82	0.66	0.066
	(ref.)	(0.72–2.31)	(0.43–1.55)	(0.34–1.27)	
Model 1	1.00	1.72	1.27	0.94	0.594
	(ref.)	(0.95–3.12)	(0.65–2.48)	(0.42–2.10)	
Model 2	1.00	1.83	1.43	1.05	0.803
	(ref.)	(0.96–3.48)	(0.72–2.87)	(0.42–2.60)	
Repeated measurements of diet	1.00	1.06	1.08	0.72	0.483
	(ref.)	(0.51–2.21)	(0.53–2.22)	(0.30–1.76)	

Quartiles of energy-adjusted animal protein intake					
	Q1	Q2	Q3	Q4	
**Median (g/d)**	**55.1**	**68.3**	**78.7**	**93.8**	
**All-cause mortality**					
N	4498	4497	4497	4497	***p*** ** for trend**
Deaths/person-years	129/59,947	110/59,505	102/58,436	119/56,978	
Age and sex adjusted	1.00	1.01	0.96	1.21	0.172
	(ref.)	(0.78–1.31)	(0.74–1.25)	(0.95–1.55)	
Model 1	1.00	1.00	0.92	1.13	0.470
	(ref.)	(0.77–1.31)	(0.69–1.21)	(0.87–1.46)	
Model 2	1.00	0.93	0.82	0.94	0.555
	(ref.)	(0.71–1.22)	(0.61–1.09)	(0.70–1.26)	
Repeated measurements of diet	1.00 (ref.)	0.82 (0.62–1.07)	0.76 (0.57–1.01)	0.83 (0.63–1.11)	0.187
**Cancer deaths**					
N	4431	4451	4438	4436	***p*** ** for trend**
Deaths/person-years	62/59,165	64/58,939	43/57,784	58/56,228	
Age and sex adjusted	1.00	1.24	0.86	1.26	0.467
	(ref.)	(0.87–1.76)	(0.58–1.27)	(0.88–1.80)	
Model 1	1.00	1.18	0.80	1.20	0.668
	(ref.)	(0.83–1.69)	(0.54–1.21)	(0.83–1.73)	
Model 2	1.00	1.05	0.69	0.95	0.486
	(ref.)	(0.73–1.52)	(0.45–1.05)	(0.62–1.44)	
Repeated measurements of diet	1.00	0.79	0.65	0.69	0.051
	(ref.)	(0.55–1.14)	(0.43–0.97)	(0.46–1.04)	
**Cardiovascular death**					
N	4396	4399	4419	4401	***p*** ** for trend**
Deaths/person-years	27/58,831	12/58,384	24/57,550	23/55,820	
Age and sex adjusted	1.00	0.62	1.02	1.20	0.389
	(ref.)	(0.32–1.23)	(0.59–1.78)	(0.69–2.07)	
Model 1	1.00	0.58	0.83	1.00	0.887
	(ref.)	(0.29–1.18)	(0.45–1.53)	(0.57–1.74)	
Model 2	1.00	0.58	0.82	1.04	0.801
	(ref.)	(0.28–1.21)	(0.43–1.57)	(0.55–1.96)	
Repeated measurements of diet	1.00	0.67	0.66	1.09	0.842
	(ref.)	(0.33–1.35)	(0.33–1.31)	(0.59–2.01)	

Adjusted for age (underlying variable) and sex (dichotomous) stratified by deciles of age and recruitment period (5 categories). **Model 1**: additionally adjusted for alcohol intake (g/d, continuous), BMI (kg/m^2^, linear and quadratic terms, continuous), years of university education (three categories), marital status (married, others), smoking status (three categories), cumulative smoking habit (packs-years, four categories), physical activity (continuous), total energy intake (five categories), following a special diet (dichotomous), snacking (dichotomous), Trichopoulou MeDiet score (three categories), prevalent hypertension (dichotomous) and prevalent hypercholesterolemia (dichotomous). **Model 2**: additionally adjusted for animal protein (g/d, continuous) when the assessed exposure was plant protein, plant protein (g/d, continuous) when the assessed exposure was animal protein, monounsaturated fatty acids (g/d, continuous), polyunsaturated fatty acids (g/d, continuous), saturated fatty acids (g/d, continuous) and trans fatty acids (g/d, continuous).

Table 3 shows HRs and 95% CIs for all-cause mortality and cause specific mortality, according to plant and animal protein intake. Participants in the highest quartile of plant protein intake had a 45% lower hazard of all-cause mortality compared with those in the lowest quartile after adjustment for sex and age [HR: 0.55 (95% CI: 0.42–0.72)]. Results was similar in the model adjusted for sociodemographic, lifestyle risk factors and prevalent diseases at baseline (model 1) with a HR of 0.63 (95% CI: 0.46–0.86). In the fully adjusted model (model 2) individuals with higher plant protein intake (Q4) have a 35% lower risk of all-cause mortality [HR: 0.65 (95% CI: 0.45–0.93)] compared to the lowest category (Q1) and there was a linear dose-response relationship across quartiles (*p* for trend = 0.009). Similar results were found in repeated measurements [HR: 0.67 (95% CI: 0.47–0.96); *p* for trend = 0.012], with a significant inverse dose-response relation between plant protein intake and all-cause mortality.

In the age and sex adjusted model and model 1, greater plant protein intake (Q4) was associated with a lower risk of cancer mortality with an HR of 0.52 (95% CI: 0.35–0.77) compared to lower intake (Q1) and 0.61 (95% CI: 0.38–0.97), respectively. However, after adjusting for animal protein intake and intake of monounsaturated fatty acids, polyunsaturated fatty acids, saturated fatty acids and trans-fatty acids (model 2), the association became non-significant with an HR of 0.67 (95% CI: 0.40–1.13). Also, a borderline significant association was found for repeated measurements [HR Q4 vs. Q1: 0.60 (95% CI: 0.37–1.00); *p* for trend = 0.014]. Moreover, there was no statistically significant association between highest plant protein intake and cardiovascular disease mortality [HR Q4 vs. Q1: 1.05 (95% CI: 0.42–2.60); *p* for trend = 0.803] compared to the lowest intake.

No significant association was found between animal protein intake and all-cause mortality after adjustment for sex and age [HR: 1.21 (95% CI: 0.95–1.55)] and after multivariate adjustments [HR: 0.94 (95% CI: 0.70–1.26)].

**Fig. 1:**
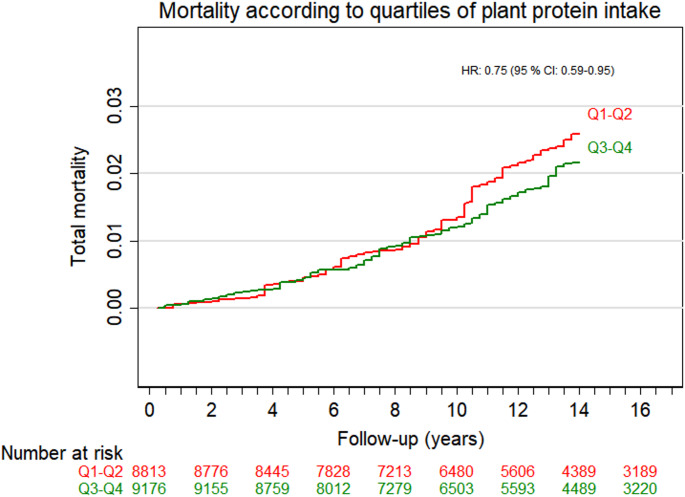
Nelson-Aalen plot for rates of mortality during follow-up according to baseline plant protein intake (g/d). The estimates were adjusted for sex, age, alcohol intake (g/d, continuous), BMI (kg/m^2^, linear and quadratic terms, continuous), years of university education (three categories), marital status (married, others), smoking status (three categories), cumulative smoking habit (packs-years, four categories), physical activity (continuous), total energy intake (five categories), following a special diet (dichotomous), snacking (dichotomous), Trichopoulou MeDiet score (three categories), prevalent hypertension (dichotomous), prevalent hypercholesterolemia (dichotomous), animal protein (g/d, continuous), monounsaturated fatty acids (g/d, continuous), polyunsaturated fatty acids (g/d, continuous), saturated fatty acids (g/d, continuous) and trans fatty acids (g/d, continuous) with the use of inverse probability weighting

**Fig. 2:**
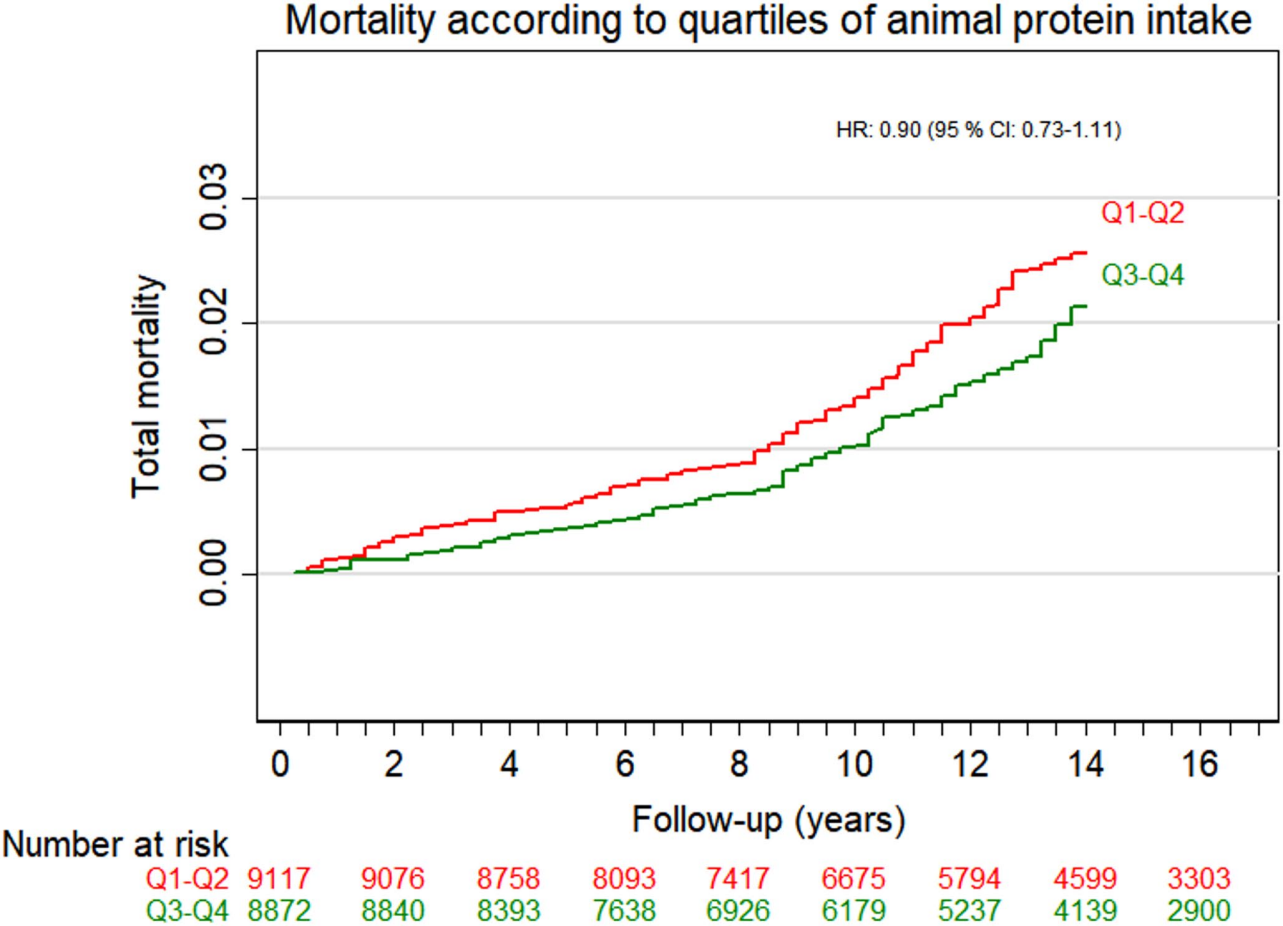
Nelson-Aalen plot for rates of mortality during follow-up according to baseline animal protein intake (g/d). The estimates were adjusted for sex, age, alcohol intake (g/d, continuous), BMI (kg/m², linear and quadratic terms, continuous), years of university education (three categories), marital status (married, others), smoking status (three categories), cumulative smoking habit (packs-years, four categories), physical activity (continuous), total energy intake (five categories), following a special diet (dichotomous), snacking (dichotomous), Trichopoulou MeDiet score (three categories), prevalent hypertension (dichotomous), prevalent hypercholesterolemia (dichotomous), plant protein (g/d, continuous), monounsaturated fatty acids (g/d, continuous), polyunsaturated fatty acids (g/d, continuous), saturated fatty acids (g/d, continuous) and trans fatty acids (g/d, continuous) with the use of inverse probability weighting

Rates of mortality during follow-up according to baseline plant protein intake (g/d) and animal protein intake (g/d) are described in Figs. 1 and 2, after applying inverse probability weighting to adjust for potential confounders.

In substitution analyses, we did not find a significant association for the isocaloric replacement of plant protein with animal protein [HR: 0.83 (95% CI: 0.50–1.38)], fat [HR: 0.81 (95% CI: 0.49–1.34)] or carbohydrate [HR: 0.90 (95% CI: 0.52–1.55)].

**Fig. 3:**
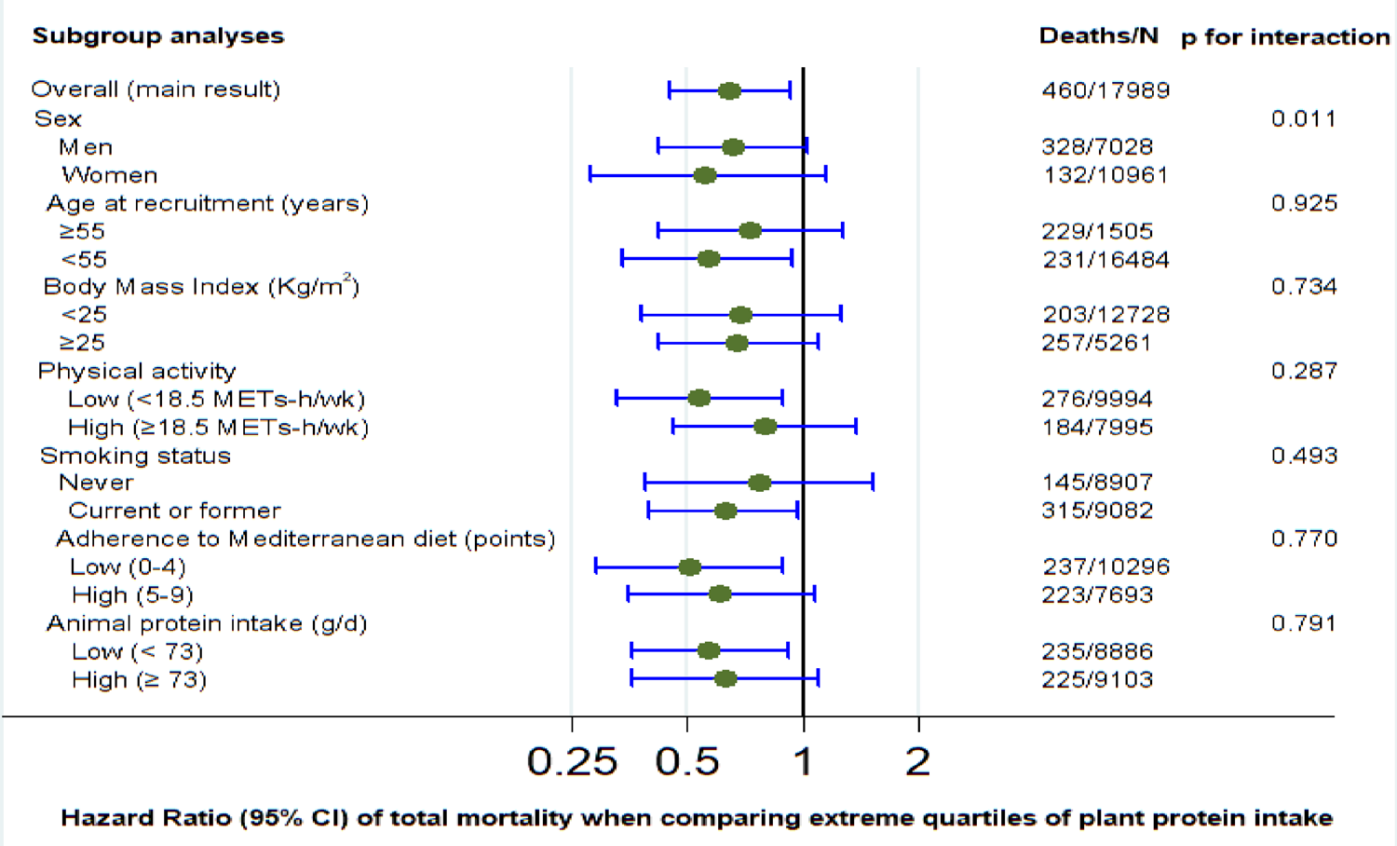
Subgroup analysis for association between plant protein intake and all-cause mortality (highest versus lowest quartile of intake). Adjusted for age (underlying variable) and sex (dichotomous) stratified by deciles of age and recruitment period (5 categories). Additionally adjusted for alcohol intake (g/d, continuous), BMI (kg/m², linear and quadratic terms, continuous), years of university education (three categories), marital status (married, others), smoking status (three categories), cumulative smoking habit (packs-years, four categories), physical activity (continuous), total energy intake (five categories), following a special diet (dichotomous), snacking (dichotomous), Trichopoulou MeDiet score (three categories), prevalent hypertension (dichotomous), prevalent hypercholesterolemia (dichotomous), animal protein (g/d, continuous), monounsaturated fatty acids (g/d, continuous), polyunsaturated fatty acids (g/d, continuous), saturated fatty acids (g/d, continuous) and trans fatty acids (g/d, continuous)

We conducted subgroup analyses by repeating the Cox regression models in different scenarios comparing the fourth quartile with the first quartile of plant protein intake (Fig. 3 and Supplemental Table 1). The interaction with sex was statistically significant (*p* < 0.011). The HR for men was 0.66 (95% CI: 0.42–1.03) while for women was 0.56 (95% CI: 0.28–1.15). The rest of the interactions were not significant. Additionally, we calculated *p* for interactions between animal protein intake and stratification variables (Supplemental Fig. 2 and Supplemental Table 2). However, we did not find any statistically significant interaction.

Additionally, we evaluated the association between animal protein intake and all-cause mortality according to specific dietary sources. No statistically significant associations were observed between protein intake from red meat, white meat, fish, eggs, or dairy products and all-cause mortality (Supplementary Table 3).

Moreover, we calculated the E value proposed by VanderWeele et al. In our study, the E value was 2.45 for the estimate and 1.360 for the CI. The observed HR of 0.65 in our analysis could be explained by the presence of an unmeasured confounder associated with both plant protein intake and mortality would need a minimum HR of 2.45 to fully explain the observed association. However, the presence of such a confounder cannot be ruled out. Similarly, the lowest CI could be adjusted to include the null due to an unmeasured confounder linked to both plant protein intake and mortality, each with an HR of 1.36, in addition to the measured confounders. Nevertheless, a confounder with a lesser effect would not sufficiently explain this result.

**Fig. 4:**
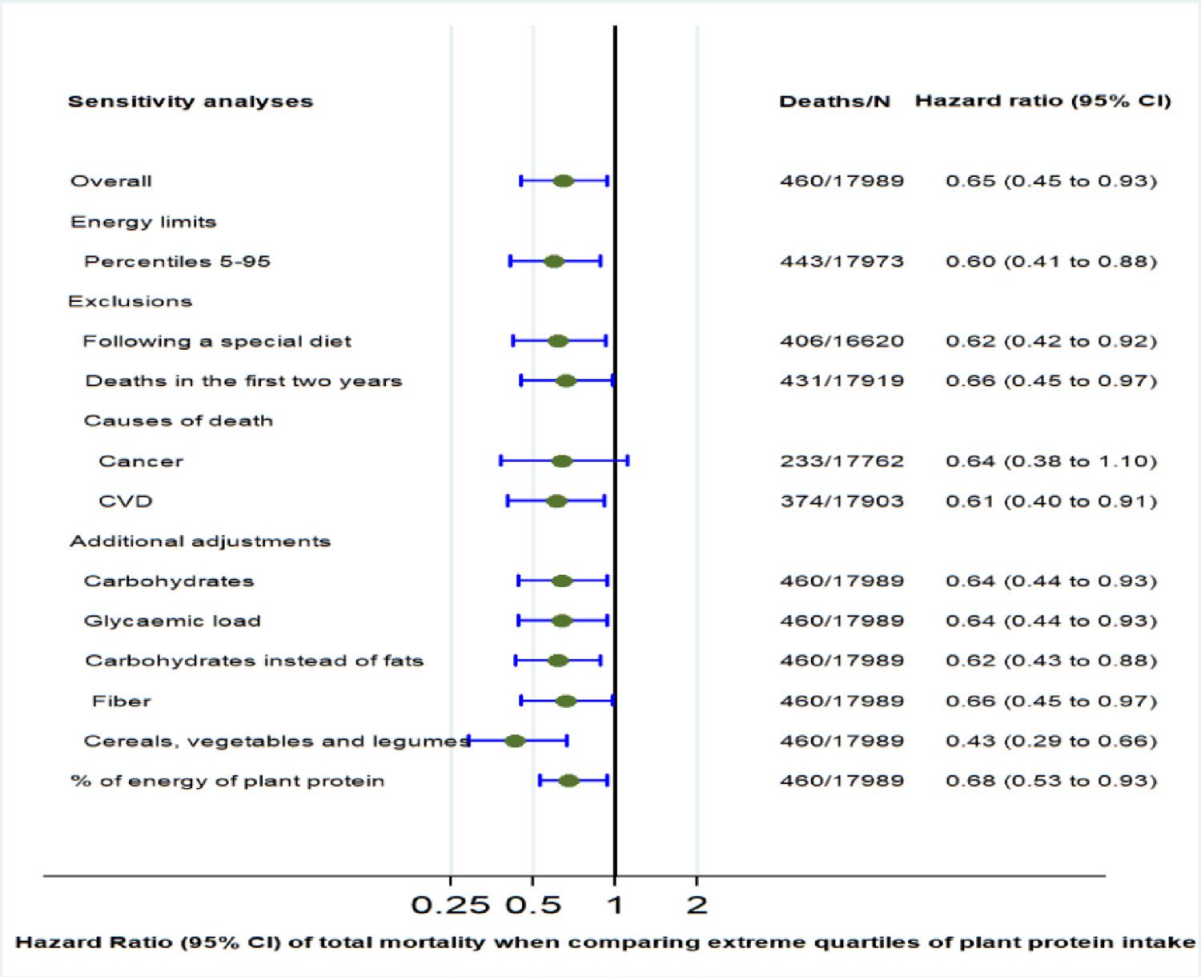
Sensitivity analyses for the association between plant protein intake and all-cause mortality (highest versus lowest quartile of intake). Adjusted for age (underlying variable) and sex (dichotomous) stratified by deciles of age and recruitment period (5 categories). Additionally adjusted for alcohol intake (g/d, continuous), BMI (kg/m², linear and quadratic terms, continuous), years of university education (three categories), marital status (married, others), smoking status (three categories), cumulative smoking habit (packs-years, four categories), physical activity (continuous), total energy intake (five categories), following a special diet (dichotomous), snacking (dichotomous), Trichopoulou MeDiet score (three categories), prevalent hypertension (dichotomous), prevalent hypercholesterolemia (dichotomous), animal protein (g/d, continuous), monounsaturated fatty acids (g/d, continuous), polyunsaturated fatty acids (g/d, continuous), saturated fatty acids (g/d, continuous) and trans fatty acids (g/d, continuous)

The sensitivity analyses comparing the highest quartile with the lowest quartile of plant protein intake are shown in Fig. 4. The association between plant protein intake and all-cause mortality persisted in different scenarios suggesting that the association between higher plant protein intake and all-cause mortality is robust. Furthermore, no associations were found between higher animal protein intake and all-cause mortality in the sensitivity analyses (Supplemental Fig. 3).

## Discussion

In a Mediterranean population of relatively young Spanish subjects, those individuals with a higher plant protein intake showed a 35% lower risk of all-cause mortality compared to those with lower intake. In contrast, animal protein intake showed no significant association with all-cause mortality. Similar results were observed in repeated measurements.

### Comparison with other studies

These results support the findings previously published in several meta-analyses [14, 29, 30]. The meta-analyses performed by Qi X et al. showed an inverse relationship between a higher plant protein intake and all-cause mortality [Relative risk (RR): 0.92 (95% CI: 0.88–0.96)] [29]. Nevertheless, other studies did not find an association [13–15, 31, 32]. In the Rotterdam study [14], no significant association between plant protein intake and all-cause mortality was found [HR: 1.06 (95% CI: 0.92–1.21)]. Besides, in our study, plant protein intake was expressed as g/d adjusted for total energy intake using the residuals method [26]. However, in most studies, it was expressed as % of energy from plant protein intake to total energy intake [10–14, 33, 34], making comparability between studies difficult. Additionally, we performed a sensitivity analysis in which plant protein intake was expressed as % of energy from plant protein intake to total energy intake and the result was quite similar.

Our results regarding animal protein intake are in agreement with results obtained in previous meta-analysis [14, 29, 30]. In a meta-analysis including a total of 304,100 subjects and 60,495 deaths, Naghshi et al. [30] found that animal protein intake was not significantly associated with all-cause mortality. However, in other prospective studies conducted in Europe [13, 14, 31] and Australia [35] a higher animal protein intake was significantly associated with all-cause mortality. The inconsistency in findings between studies may be related to differences in the main sources of animal protein intake. Animal protein includes different protein sources that may have different effects on mortality. Unprocessed and processed red meat have been linked to an increased risk of mortality [36–39], while dairy products show no association [40, 41]. In contrast, fish and white meat are associated with a lower risk of death [36, 42, 43]. The absence of significant differences may be attributed to the fact that two of the primary sources of animal protein in our study were dairy products (27.0%) and fish and seafood (18.5%).

In the SUN cohort, the mean protein intake was 18.3% (SD = 3.3%), slightly above the Spanish population’s 16.8% according to the ANIBES study, a representative sample of the Spanish population [44].

In our study 30% of the protein intake came from plant protein intake and 70% from animal protein intake as in the studies of Sun et al. [34] and Virtanen et al. [31] conducted in an American and a Finnish population. Other European [14] and American [10, 16] studies showed plant protein intake at 20–40% and animal protein at 60–80%, while Asian studies reported around 50% for both [12, 33].

The median percentage of energy from animal protein and plant protein to total energy intake of the highest category in our cohort was 16.6% and 6.8%, respectively. In the Rotterdam study [14], a population-based cohort in the Netherlands that included 7786 subjects, the median animal protein intake in the highest quartile was slightly lower (13.4%) than in ours, while the median plant protein intake was similar (7.3%). The PREDIMED study [13], which included 7216 older Spanish participants at high cardiovascular risk, described a median of animal protein intake of 13.90% for the highest category which was lower than our study, probably due to the age differences between populations. However, the median of plant protein intake was comparable to ours (6.6%). In other studies, the amounts of animal and plant protein in the highest category were very similar [10, 11, 34] to the SUN cohort, except for the Asian studies where the amount of animal protein was lower and the amount of plant protein was higher than the rest of the studies [12, 33]. Diets in Asian countries are characterised by a higher consumption of plant-based foods compared to those in America or Europe, which may explain their lower intake of animal protein.

### Mechanisms

In the SUN cohort the main sources of plant protein were cereals (33.4%), followed by vegetables (32.2%) and then legumes (15.3%). Previous studies found an inverse association between whole grain cereal and cereal fiber intake and all-cause mortality [45]. Additionally, other plant protein sources like vegetables and legumes, which are the second and third sources of plant protein in our cohort, have been linked to lower mortality [46–49]. Additionally, plant protein has a lower concentration of essential amino acids such as methionine or lysine and a higher concentration of non-essential amino acids such as glutamine, arginine and glycine which have been associated with lower cardiovascular risk [8, 50, 51]. Glutamine may improve glucose metabolism and insulin sensitivity while reducing oxidative stress and inflammation [52]; arginine, as a nitric oxide precursor, promotes vasodilation and may contribute to blood pressure reduction [53]; and glycine may modulate insulin resistance through glutathione synthesis regulation [54]. Another potential mechanism may involve the gut microbiota, as plant protein-rich diets have been associated with increased abundance of anti-inflammatory butyrate-producing bacteria [55].In addition, plant proteins are low in branched-chain and aromatic amino acids, associated with an increased risk of cardiovascular disease [56, 57].

Plant protein is not consumed in isolation; it is consumed together with other nutrients such as polyunsaturated fatty acids, fiber, minerals or vitamins. It may be that the beneficial effect is not only due to the vegetable protein itself, but also to the other nutrients that it contains [58]. This possibility is supported by our isocaloric substitution analyses, which did not show a significant protective effect when plant protein replaced animal protein, fat, or carbohydrate; therefore, our results suggest that the observed benefit may reflect other nutrients or overall dietary patterns rather than an independent effect of plant protein itself.

### Limitations and strengths of this study

Our study had several limitations that should be acknowledged. First, we used a self-reported FFQ to assess protein intake, so it is possible to have some degree of measurement error. Nevertheless, it is considered the most appropriate method to evaluate nutrient intake in large observational studies and have been previously validated and re-validated in Spain [17–19]. Participants are also highly educated, which improves the quality of self-reported information, reducing the potential confounding related to educational level and increasing the internal validity of our results [59]. Second, as in all observational studies, residual confounding by unidentified confounders is still possible. However, we adjusted for a large range of confounders, and the findings remained consistent. Additionally, the E values for the point estimate supported the association between plant protein intake and all-cause mortality. The point estimate could only be theoretically explained by an unmeasured confounder with an HR of at least 2.45-fold for all-cause mortality and plant protein intake. Third, our sample was not representative of the general population because they are relatively young university graduates, with a relatively low prevalence of risk factors for chronic diseases. This limits the external validity of the findings, and these results are not applicable to the general population, to elderly people or to patients with diseases. However, the generalizability of the findings should be based on biological plausibility. Four, protein is not eaten in isolation but is consumed together with other nutrients such as iron, saturated fatty acids, polyunsaturated fatty acids or fiber which may have different effects on mortality. However, in this study, we adjusted for different types of fatty acids, and we carried out sensitivity analyses adjusting for fiber and the results remained statistically significant.

The major strengths of the study rely on its large sample size, its prospective design with long follow-up time, its high response rate and the adjustment for a wide number of potential confounders and the confirmation of mortality cases by medical records or consultation of the National Death Index. Moreover, we performed multiple sensitivity analyses and repeated measurements, as most studies have only assessed protein intake at baseline, which adds robustness to our results. In addition, most studies examining the association between protein intake and mortality have been performed in populations whose average age is higher than ours or at high risk of chronic diseases.

## Conclusion

Plant protein was inversely associated with total mortality in a Mediterranean population. These results support public health recommendations to increase the intake of plant proteins such as proteins from grains, legumes or vegetables to prevent all-cause mortality in the context of a Mediterranean population. Further studies are needed to assess the role of animal protein intake on mortality in a relatively young Mediterranean population, as well as studies that perform repeated measurements of protein intake. However, plant protein intake was not statistically associated with cancer mortality or CVD mortality after adjusting for multiple confounding factors.

## Supplementary Information

Below is the link to the electronic supplementary material.

Supplementary Material 1

## Data Availability

The information from the SUN Project that backs up our findings can be requested from the Department of Preventive Medicine and Public Health, School of Medicine, University of Navarra (Spain) at sun@unav.es .
