# Supplementary material for: High plant protein intake decreases all-cause mortality in the “Seguimiento Universidad de Navarra” (SUN) cohort European journal of nutrition
Source: Eur J Nutr. 2026 Aug 1;65(5):226. doi: 10.1007/s00394-026-04037-0 (PMC13428695; doi:10.1007/s00394-026-04037-0)
Supplement: Supplementary file 1 — Supplementary Material 1 [file 394_2026_4037_MOESM1_ESM.docx]

**Supplemental material**

**High plant protein intake decreases all-cause mortality in the “Seguimiento Universidad de Navarra” (SUN) cohort**

***European Journal of Nutrition***

Ainara Martínez-Tabar **^1,2^**, Miguel Ruiz-Canela **^1,2,3^**, Vanessa Bullón-Vela **^1,2^**, Carmen de la Fuente-Arrillaga **^1,2,3^**, Carmen Sayón-Orea **^1,2,3^**, Miguel Ángel Martínez-González **^1,2,3^**, Maira Bes-Rastrollo **^1,2,3,*^**

**^1^**University of Navarra, Department of Preventive Medicine and Public Health, Pamplona, Spain

**^2^**IdiSNA (Navarra Medical Research Institute), Pamplona, Spain

**^3^**CIBER Physiopathology of Obesity and Nutrition (CIBEROBN), Madrid, Spain

*Maira Bes Rastrollo

[mbes@unav.es](mailto:mbes@unav.es)

**Supplemental Figure 1. Flow-chart of the study participants in the SUN cohort study.**

**n=1,379**

Lost to follow-up

(Retention rate: 93%)

**n=220**

Participants with no answer in > = 70 items of the FFQ

**n=1,188**

Participants with prevalent CVD, cancer and T2DM at baseline

**n=2,123**

Outside of predefined limits for total energy intake

**n=234**

Participants with insufficient follow-up time

Participants recruited before May 2022

**n=23,133**

**Abbreviations:** T2DM: Type 2 diabetes.

**n=22,899**

Participants

**n= 17,989**

Participants

**n= 19,368**

Participants

**n= 19,588**

Participants

**n= 20,776**

Participants

**
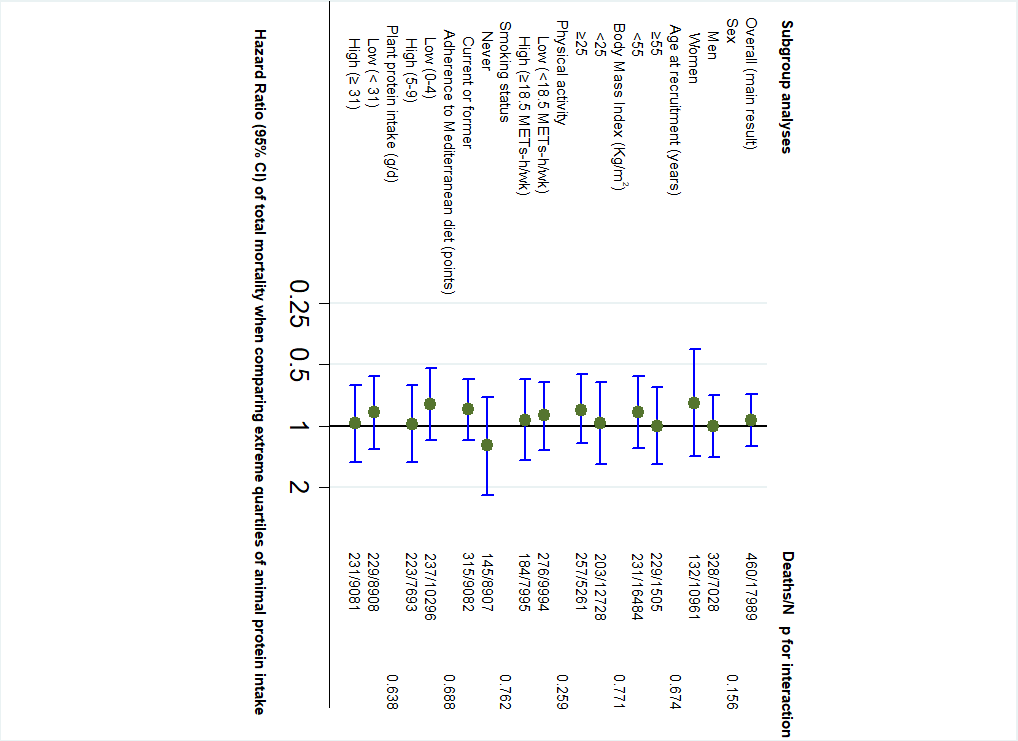
**

**Supplemental Figure 2. Subgroup analysis for association between animal protein intake and all-cause mortality (highest versus lowest quartile of intake).** Adjusted for age (underlying variable) and sex (dichotomous) stratified by deciles of age and recruitment period (5 categories). Additionally adjusted for alcohol intake (g/d, continuous), BMI (kg/m² , linear and quadratic terms, continuous), years of university education (three categories), marital status (married, others), smoking status (three categories), cumulative smoking habit (packs-years, four categories), physical activity (continuous), total energy intake (five categories), following a special diet (dichotomous), snacking (dichotomous), Trichopoulou MeDiet score (three categories), prevalent hypertension (dichotomous), prevalent hypercholesterolemia (dichotomous), plant protein (g/d, continuous), monounsaturated fatty acids (g/d, continuous), polyunsaturated fatty acids (g/d, continuous), saturated fatty acids (g/d, continuous) and trans fatty acids (g/d, continuous).

**
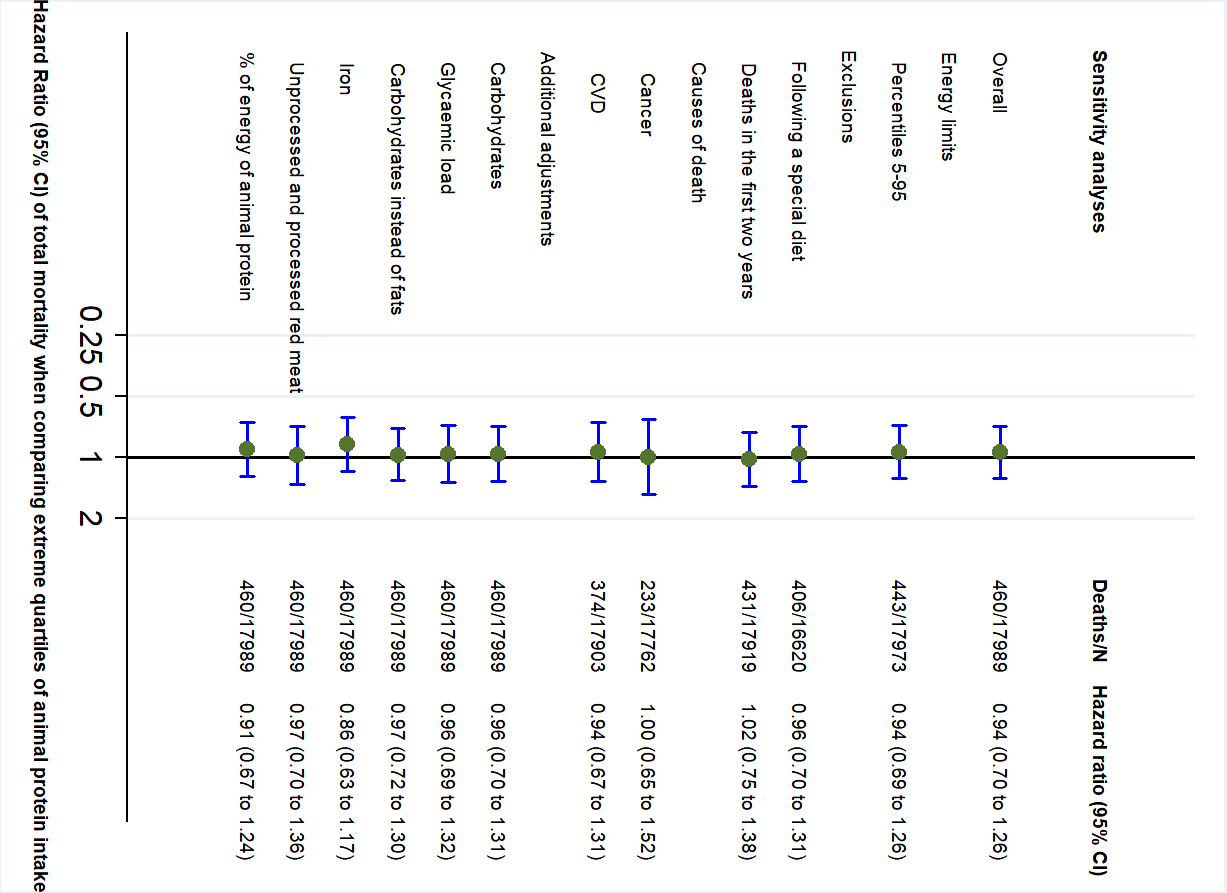
**

**Supplemental Figure 3. Sensitivity analyses for the association between animal protein intake and all-cause mortality (highest versus lowest quartile of intake).** Adjusted for age (underlying variable) and sex (dichotomous) stratified by deciles of age and recruitment period (3 categories). Additionally adjusted for alcohol intake (g/d, continuous), BMI (kg/m² , linear and quadratic terms, continuous), years of university education (three categories), marital status (married, others), smoking status (three categories), cumulative smoking habit (packs-years, four categories), physical activity (continuous), total energy intake (five categories), following a special diet (dichotomous), snacking (dichotomous), Trichopoulou MeDiet score (three categories), prevalent hypertension (dichotomous), prevalent hypercholesterolemia (dichotomous), plant protein (g/d, continuous), monounsaturated fatty acids (g/d, continuous), polyunsaturated fatty acids (g/d, continuous), saturated fatty acids (g/d, continuous) and trans fatty acids (g/d, continuous).

**Supplemental Table 1. Subgroup analyses for association between plant protein intake and all-cause mortality according to quartiles of plant protein intake.**

|  | **Hazard ratio (95% CI)** | **p for interaction** |
| --- | --- | --- |
| **Overall** | Q1: 1.00 (ref.)  Q2: 0.99 (0.75-1.31)  Q3: 0.81 (0.60-1.11)  Q4: 0.65 (0.45-0.93) |  |
| **Subgroup analyses** |  |  |
| **Sex** |  | p=0.011 |
| Men | Q1: 1.00 (ref.)  Q2: 0.77 (0.54-1.08)  Q3: 0.81 (0.56-1.17)  Q4: 0.66 (0.42-1.03) |  |
| Women | Q1: 1.00 (ref.)  Q2: 1.57 (0.95-2.58)  Q3: 0.82 (0.45-1.49)  Q4: 0.56 (0.28-1.15) |  |
| **Age at recruitment(years)** |  | p=0.925 |
| ≥ 55 | Q1: 1.00 (ref.)  Q2: 1.11 (0.73-1.71)  Q3: 0.88 (0.54-1.41)  Q4: 0.73 (0.42-1.27) |  |
| <55 | Q1: 1.00 (ref.)  Q2: 0.89 (0.61-1.30)  Q3: 0.75 (0.49-1.15)  Q4: 0.57 (0.34-0.94) |  |
| **Body mass index (kg/m^2^)** |  | p=0.734 |
| <25 | Q1: 1.00 (ref.)  Q2: 1.15 (0.74-1.79)  Q3: 0.80 (0.47-1.35)  Q4: 0.69 (0.38-1.26) |  |
| ≥ 25 | Q1: 1.00 (ref.)  Q2: 0.87 (0.60-1.25)  Q3: 0.84 (0.56-1.26)  Q4: 0.68 (0.42-1.10) |  |
| **Physical activity** |  | p=0.287 |
| Low (<18.5 METs-h/wk) | Q1: 1.00 (ref.)  Q2: 0.94 (0.66-1.35)  Q3: 0.84 (0.56-1.26)  Q4: 0.54 (0.33-0.89) |  |
| High (≥18.5 METs-h/wk) | Q1: 1.00 (ref.)  Q2: 1.22 (0.76-1.94)  Q3: 0.84 (0.50-1.40)  Q4: 0.80 (0.46-1.38) |  |
| **Smoking status** |  | p= 0.493 |
| Never | Q1: 1.00 (ref.)  Q2: 1.16 (0.67-2.01)  Q3: 0.70 (0.38-1.27)  Q4: 0.77 (0.39-1.52) |  |
| Current or former | Q1: 1.00 (ref.)  Q2: 0.96 (0.70-1.33)  Q3: 0.89 (0.61-1.29)  Q4: 0.63 (0.40-0.97) |  |
| **Mediterranean diet (points)** |  | p=0.770 |
| High adherence (5-9) | Q1: 1.00 (ref.)  Q2: 0.84 (0.48-1.46)  Q3: 0.76 (0.44-1.29)  Q4: 0.61 (0.35-1.08) |  |
| Low adherence (0-4) | Q1: 1.00 (ref.)  Q2: 0.95 (0.69-1.31)  Q3: 0.71 (0.47-1.06)  Q4: 0.51 (0.29-0.89) |  |
| **Animal protein** intake (g/d) |  | p=0.791 |
| high (≥ 73) | Q1: 1.00 (ref.)  Q2: 1.05 (0.73-1.51)  Q3: 0.80 (0.50-1.28)  Q4: 0.63 (0.36-1.10) |  |
| low (< 73) | Q1: 1.00 (ref.)  Q2: 0.82 (0.53-1.27)  Q3: 0.69 (0.44-1.09)  Q4: 0.57 (0.36-0.92) |  |

Adjusted for age (underlying variable) and sex (dichotomous) stratified by deciles of age and recruitment period (5 categories). Additionally adjusted alcohol intake (g/d, continuous), BMI (kg/m², linear and quadratic terms, continuous), years of university education (three categories), marital status (married, others), smoking status (three categories), cumulative smoking habit (packs-years, four categories), physical activity (continuous), total energy intake (five categories), following a special diet (dichotomous), snacking (dichotomous), Trichopoulou MeDiet score (three categories), prevalent hypertension (dichotomous), prevalent hypercholesterolemia (dichotomous), animal protein (g/d, continuous), monounsaturated fatty acids (g/d, continuous), polyunsaturated fatty acids (g/d, continuous), saturated fatty acids (g/d, continuous) and trans fatty acids (g/d, continuous).

**Supplemental Table 2. Subgroup analyses for association between animal protein intake and all-cause mortality according to quartiles of animal protein intake.**

|  | **Hazard ratio (95% CI)** | **p for interaction** |
| --- | --- | --- |
| **Overall** | Q1: 1.00 (ref.)  Q2: 0.93 (0.71-1.22)  Q3: 0.82 (0.61-1.09)  Q4: 0.94 (0.70-1.26) |  |
| **Subgroup analyses** |  |  |
| **Sex** |  | p=0.156 |
| Men | Q1: 1.00 (ref.)  Q2: 0.84 (0.60-1.18)  Q3: 0.77 (0.55-1.10)  Q4: 1.00 (0.71-1.42) |  |
| Women | Q1: 1.00 (ref.)  Q2: 1.09 (0.67-1.78)  Q3: 0.87 (0.50-1.51)  Q4: 0.77 (0.42-1.41) |  |
| **Age at recruitment (years)** |  | p=0.674 |
| ≥ 55 | Q1: 1.00 (ref.)  Q2: 0.97 (0.65-1.45)  Q3: 0.72 (0.47-1.12)  Q4: 1.00 (0.65-1.54) |  |
| <55 | Q1: 1.00 (ref.)  Q2: 0.86 (0.59-1.24)  Q3: 0.87 (0.59-1.28)  Q4: 0.86 (0.57-1.29) |  |
| **Body mass index (kg/m^2^)** |  | p=0.771 |
| <25 | Q1: 1.00 (ref.)  Q2: 0.92 (0.62-1.38)  Q3: 0.99 (0.64-1.54)  Q4: 0.97 (0.61-1.54) |  |
| ≥ 25 | Q1: 1.00 (ref.)  Q2: 0.87 (0.61-1.28)  Q3: 0.69 (0.46-1.02)  Q4: 0.84 (0.56-1.22) |  |
| **Physical activity** |  | p=0.259 |
| Low (<18.5 METs-h/wk) | Q1: 1.00 (ref.)  Q2: 0.99 (0.71-1.38)  Q3: 0.71 (0.48-1.05)  Q4: 0.89 (0.61-1.32) |  |
| High (≥18.5 METs-h/wk) | Q1: 1.00 (ref.)  Q2: 0.81 (0.50-1.30)  Q3: 0.94 (0.58-1.52)  Q4: 0.94 (0.59-1.48) |  |
| **Smoking status** |  | P= 0.762 |
| Never | Q1: 1.00 (ref.)  Q2: 1.32 (0.82-2.13)  Q3: 0.93 (0.54-1.61)  Q4: 1.25 (0.72-2.18) |  |
| Current or former | Q1: 1.00 (ref.)  Q2: 0.82 (0.60-1.14)  Q3: 0.80 (0.57-1.13)  Q4: 0.83 (0.59-1.18) |  |
| **Mediterranean diet (points)** |  | p=0.688 |
| High adherence (5-9) | Q1: 1.00 (ref.)  Q2: 1.05 (0.73-1.52)  Q3: 0.83 (0.54-1.27)  Q4: 0.98 (0.63-1.51) |  |
| Low adherence (0-4) | Q1: 1.00 (ref.)  Q2: 0.78 (0.53-1.14)  Q3: 0.73 (0.49-1.09)  Q4: 0.78 (0.52-1.17) |  |
| **Plant protein intake (g/d)** |  | p=0.638 |
| High (≥ 31) | Q1: 1.00 (ref.)  Q2: 0.90 (0.62-1.30)  Q3: 0.96 (0.65-1.41)  Q4: 0.97 (0.63-1.50) |  |
| low (< 31) | Q1: 1.00 (ref.)  Q2: 0.94 (0.61-1.46)  Q3: 0.74 (0.48-1.15)  Q4: 0.86 (0.57-1.30) |  |

Adjusted for age (underlying variable) and sex (dichotomous) stratified by deciles of age and recruitment period (5 categories). Additionally adjusted alcohol intake (g/d, continuous), BMI (kg/m², linear and quadratic terms, continuous), years of university education (three categories), marital status (married, others), smoking status (three categories), cumulative smoking habit (packs-years, four categories), physical activity (continuous), total energy intake (five categories), following a special diet (dichotomous), snacking (dichotomous), Trichopoulou MeDiet score (three categories), prevalent hypertension (dichotomous), prevalent hypercholesterolemia (dichotomous), plant protein (g/d, continuous), monounsaturated fatty acids (g/d, continuous), polyunsaturated fatty acids (g/d, continuous), saturated fatty acids (g/d, continuous) and trans fatty acids (g/d, continuous).

**Supplementary Table 3. Cox proportional hazard ratios (HRs) and 95% confidence intervals (CIs) for all-cause mortality according to animal protein intake, stratified by food sources.**

|  | **Q1** | | **Q2** | **Q3** | **Q4** |
| --- | --- | --- | --- | --- | --- |
|  |  | | **Hazard ratio (95% CI)** | |  |
| **Protein from red meat** | | | | | |
| Age and sex adjusted | 1.00 (Ref.) | | 1.26  (0.98-1.62) | 1.25  (0.97-1.62) | 1.09  (0.83-1.43) |
| Model 1 | 1.00 (Ref.) | | 1.18  (0.91-1.54) | 1.21  (0.92-1.60) | 0.97  (0.73-1.28) |
| Model 2 | 1.00 (Ref.) | | 1.13  (0.86-1.48) | 1.12  (0.85-1.49) | 0.84  (0.62-1.15) |
| **Protein from white meat** | | |  |  |  |
| Age and sex adjusted | | 1.00 (Ref.) | 1.15  (0.89-1.48) | 1.01  (0.77-1.33) | 1.11  (0.86-1.44) |
| Model 1 | 1.00 (Ref.) | | 1.22  (0.93-1.59) | 1.07  (0.80-1.42) | 1.07  (0.82-1.40) |
| Model 2 | 1.00 (Ref.) | | 1.20  (0.92-1.56) | 1.06  (0.80-1.40) | 1.05  (0.81-1.37) |
| **Protein from fish and seafood** | | | |  |  |
| Age and sex adjusted | | 1.00 (Ref.) | 0.86  (0.64-1.14) | 0.87  (0.65-1.16) | 0.93  (0.71-1.22) |
| Model 1 | 1.00 (Ref.) | | 0.82  (0.61-1.10) | 0.90  (0.67-1.23) | 0.91  (0.76-1.34) |
| Model 2 | 1.00 (Ref.) | | 0.81  (0.60 -1.09) | 0.87  (0.64-1.18) | 0.95  (0.71-1.27) |
| **Protein from eggs** | | |  |  |  |
| Age and sex adjusted | | 1.00 (Ref.) | 0.89  (0.69-1.16) | 0.83  (0.64-1.07) | 0.89  (0.70-1.14) |
| Model 1 | 1.00 (Ref.) | | 1.01  (0.74-1.38) | 0.87  (0.65-1.16) | 0.92  (0.71-1.20) |
| Model 2 | 1.00 (Ref.) | | 0.98  ( 0.72-1.34) | 0.83  (0.62-1.12 | 0.87  (0.67-1.14) |
| **Protein from dairy products** | | | 0.97  (0.75-1.26) | 0.94  (0.73-1.22) | 1.01  (0.78-1.30) |
| Model 1 | 1.00 (Ref.) | | 0.97  (0.74-1.27) | 0.97  (0.74-1.27) | 0.94  (0.78-1.37) |
| Model 2 | 1.00 (Ref.) | | 0.94  (0.72-1.24) | 0.91  (0.69-1.21) | 0.92  (0.67-1.26) |

Adjusted for age (underlying variable) and sex (dichotomous) stratified by deciles of age and recruitment period (5 categories). **Model 1:** additionally adjusted for alcohol intake (g/d, continuous), BMI (kg/m² , linear and quadratic terms, continuous), years of university education (three categories), marital status (married, others), smoking status (three categories), cumulative smoking habit (packs-years, four categories), physical activity (continuous), total energy intake (five categories), following a special diet (dichotomous), snacking (dichotomous), Trichopoulou MeDiet score (three categories), prevalent hypertension (dichotomous) and prevalent hypercholesterolemia (dichotomous). **Model 2:** additionally adjusted for animal protein (g/d, continuous) when the assessed exposure was plant protein, plant protein (g/d, continuous) when the assessed exposure was animal protein, monounsaturated fatty acids (g/d, continuous), polyunsaturated fatty acids (g/d, continuous), saturated fatty acids (g/d, continuous) and trans fatty acids (g/d, continuous). All models were additionally adjusted for all other protein sources except the exposure variable.
